# Commutative Algebra Modeling in Materials Science – A Case Study on Metal–Organic Frameworks (MOFs)

**DOI:** 10.1021/acs.jcim.5c02817

**Published:** 2026-02-17

**Authors:** Caleb Simiyu Khaemba, Hongsong Feng, Dong Chen, Chun-Long Chen, Guo-Wei Wei

**Affiliations:** † Department of Mathematics, 3078 Michigan State University, East Lansing, Michigan 48824, United States; ‡ Department of Mathematics and Statistics, 14727 University of North Carolina at Charlotte, Charlotte, North Carolina 28223, United States; § Physical Sciences Division, 6865 Pacific Northwest National Laboratory, Richland, Washington 99354, United States; ∥ Department of Electrical and Computer Engineering, Michigan State University, East Lansing, Michigan 48824, United States; ⊥ Department of Biochemistry and Molecular Biology, Michigan State University, East Lansing, Michigan 48824, United States

## Abstract

Metal–organic frameworks (MOFs) are a class of important crystalline and highly porous materials whose hierarchical geometry and chemistry hinder interpretable predictions in materials properties. Commutative algebra is a branch of abstract algebra that has been rarely applied in data and material sciences. We introduce the first ever commutative algebra modeling and prediction in materials science. Specifically, category-specific commutative algebra (CSCA) is proposed as a new framework for MOF representation and learning. It integrates element-based categorization with multiscale algebraic invariants to encode both local coordination motifs and global network organization of MOFs. These algebraically consistent, chemically aware representations enable compact, interpretable, and data efficient modeling of MOF properties such as Henry’s constants and uptake capacities for common gases. Compared to traditional geometric and graph-based approaches, CSCA achieves comparable or superior predictive accuracy while substantially improving interpretability and stability across data sets. By aligning commutative algebra with the chemical hierarchy, the CSCA establishes a rigorous and generalizable paradigm for understanding structure and property relationships in porous materials and provides a nonlinear algebra-based framework for data-driven material discovery.

## Graphical abstract

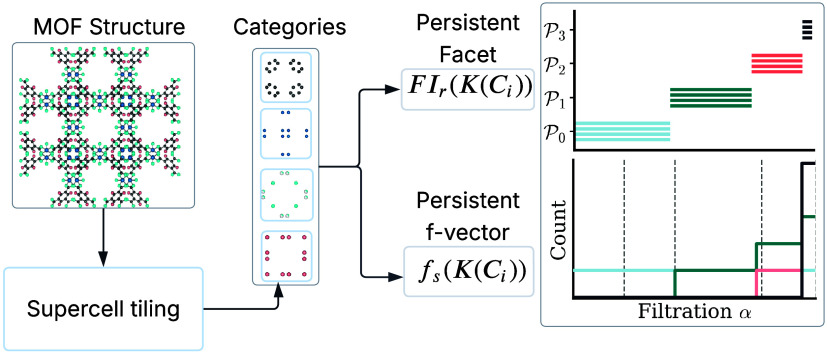

## 1 Introduction

Materials science concerns material’s structure, properties, processing, and performance. A special class of materials is metal–organic frameworks (MOFs), crystalline networks with open porosity and tunable chemistry. MOFs are attractive for adsorption, gas storage, separations, catalysis, sensing, and transport.^1–3^ They are assembled from metal nodes and organic linkers, and the way these building blocks connect creates a large design space with controllable ring sizes, pore shapes, and network connectivity that strongly influence material properties.^4–6^ Moreover, experimental studies have shown that missing-linker defects in UiO-66 can be highly tunable and can induce pronounced changes in gas adsorption behavior.^7^ This flexibility can be further enhanced by varying the choice of nodes and linkers or by applying postsynthetic treatments,^8,9^ which has enabled applications ranging from energy conversion and storage to drug delivery.^10^ An important example is polyoxometalate-based MOFs (POM-MOFs), which combine broad networks with cage-like metal oxygen clusters such that global pore connectivity and local site chemistry interact.^11^ Important adsorption and transport properties such as Henry’s constants for oxygen and nitrogen, oxygen and nitrogen uptakes, and self-diffusivities depend on both the chemistry of the cluster environment and the architecture of the pore network.^12^ Recent multiscale studies show that prediction of these adsorption and transport properties improves when chemical information is combined with geometric and topological features of the pore network,^12^ motivating our approach of modeling MOFs by linking chemical environments at the local scale with framework wide structural characteristics.

Traditional experimental characterization remains the benchmark, but scales poorly with the design space. Computational workflows broaden coverage: grand canonical Monte Carlo (GCMC) often with Widom insertion for Henry’s constant and uptake, classical molecular dynamics (MD) for diffusion, and density functional theory (DFT) for charge assignment and adsorption energetics.^13–15^ Still, sweeping conditions with GCMC, running long MD trajectories, and repeating DFT over thousands of frameworks are computationally intensive. To increase throughput, data-driven surrogates compress each framework into fixed-length features and learn a mapping to targets; for example, framework guest energy histograms with sparse regression enable rapid screening validated by simulation and experiment.^16^

Supervised machine learning (ML) benefits from standardized structures and labels in CoRE MOF, hMOF, and QMOF.^6,17,18^ Chemistry-informed priors such as HSAB (hard–soft acid–base) labels for metals and linkers, and compatibility scores can improve generalization and interpretability for stability and adsorption.^19^ Deep learning (DL) models such as convolutional neural networks (CNNs) learn directly from crystal graphs, bypassing hand crafted descriptors and linking predictions to local chemical environments.^20^ Pretrained multimodal transformers that fuse atom graph and energy grid embeddings can be fine-tuned to predict adsorption, diffusion, and electronic properties with transferable, attention-based insight.^21^ Although recent methods have captured rich MOF structures, they still struggle in giving accurate predictions for some properties. Descriptor-based machine learning underperforms for certain key MOF properties, even with energy histogram features, for example, when capturing the microenvironment of metal clusters and their interactions with substrates. Meanwhile, DL approaches (CNNs and Transformers) often entail high computational cost and substantial data requirements, constraining their utility in small data regimes.^20,21^ Complementary directions include feature driven pipelines using energy based surrogates, and geometric and chemical summaries such as energy histograms,^16^ and representation learning with graph neural networks and MOF-specific Transformers that derive features directly from structure.^21,22^ Most MOF ML studies still rely on conventional descriptors such as geometry and atom types. Recent literature, however, has highlighted topology-based alternatives that capture network connectivity and periodicity.^23–25^ Several works explore mathematically grounded constructions from algebraic topology and spectral graph theory such as topological Laplacians and persistent Laplacians for robust and high-level representations.^26–28^ However, these approaches mainly capture connectivity through spectra and persistence, and they do not encode the underlying algebraic structure or its evolution across scales.

A natural next step is to explore more fundamental and interpretable mathematical frameworks that can represent MOF structures at multiple scales. Commutative algebra, which studies commutative rings, ideals, and modules, provides such a foundation by offering algebraic tools to encode geometry and connectivity.^29,30^ Its use in data science and AI is still emerging. Persistent Stanley-Reisner theory (PSRT) bridges commutative algebra, algebraic topology, and machine learning.^31^ Classical Stanley-Reisner theory encodes a simplicial complex as a square free monomial ideal;^32,33^ PSRT tracks this structure across multiple geometric scales to yield computable, interpretable representations, such as persistent graded Betti numbers (via Hochster’s formula),^34^ persistent *f*-vectors and *h*-vectors, and persistent facet ideals with facet barcodes summarizing births and deaths.^31^ These multiscale features enable the commutative-algebra analysis of point clouds and have found great success in predicting protein–ligand binding,^35^ protein-nucleic acid binding,^34,36^ disease and mutation association,^37^ genomic and phylogenetic analysis.^38^ A comparative study has been carried out on the interpretability and representability of commutative algebra, algebraic topology, and topological spectral theory for real-world data.^39^ In general, commutative algebra approaches complement topological data analysis (TDA) and topological deep learning (TDL).^40–42^

In this work, we present the first ever commutative algebra model in materials science. We propose category-specific commutative algebra (CSCA) for the prediction of MOF properties such as gas adsorption. Our method is benchmarked against three large-scale baselines: MOFTransformer, PMTransformer, and descriptor-based models.^21,43,44^ The CSCA framework encodes category-wise information about metal nodes and organic linkers into compact and interpretable features, which serve as inputs to a simple supervised learner. Our goal is to achieve higher accuracy on Henry’s constants and uptakes for N_2_ and O_2_, while lowering computational cost compared to deep learning models and improving interpretability by tracing the predictions back to specific chemical categories.

The rest of this paper is organized as follows. Section 2 is devoted to the result and discussion. The proposed method is described in Section 3. This paper ends with a conclusion.

## 2 Results and Discussion

### 2.1 Workflow

The key steps of the category-specific commutative algebra model are outlined in an end-to-end pipeline as presented in Figure 1. A fixed supercell is used for all MOF structures to ensure consistency and fair comparison across different frameworks. Embedding each structure into the same supercell eliminates biases arising from differences in unit-cell size and lattice geometry, allowing the topological analysis to reflect intrinsic connectivity rather than cell-dependent effects. Each MOF is embedded in a cubic 64 Å × 64 Å × 64 Å supercell to provide a uniform spatial domain for α-complex filtration across all structures. Although the supercell size is set to 64 × 64 × 64 to ensure sufficient periodic representation, the topological filtration threshold used in this work is α_max_ = 12 Å, which defines the effective length scale for constructing the algebraic descriptors. Since the filtration is truncated at α_max_, all resulting descriptors depend only on local and medium-range atomic neighborhoods within this cutoff. The supercell size is therefore a nominal embedding choice and does not imply that the lattice parameters of different MOFs are strictly equal or rescaled. This design preserves material-specific periodic connectivity while ensuring that all neighborhoods relevant to the filtration are fully represented and directly comparable across structures. We then partition the atoms into element categories namely *C*_*a*_,..., *C*_*h*_, *C*_all_, where *C*_all_ represents the full set, see Table 5. The atomic categories are chosen to reflect chemically distinct roles in MOFs (metal nodes, organic backbones, heteroatoms, and functional substituents) while balancing chemical specificity and statistical robustness across the data set. Common backbone species such as C, H, O, and N are separated, while chemically similar and less frequent metals are aggregated. This preserves distinct chemical roles and mitigates sparsity in category-wise statistics.

**Figure 1:**
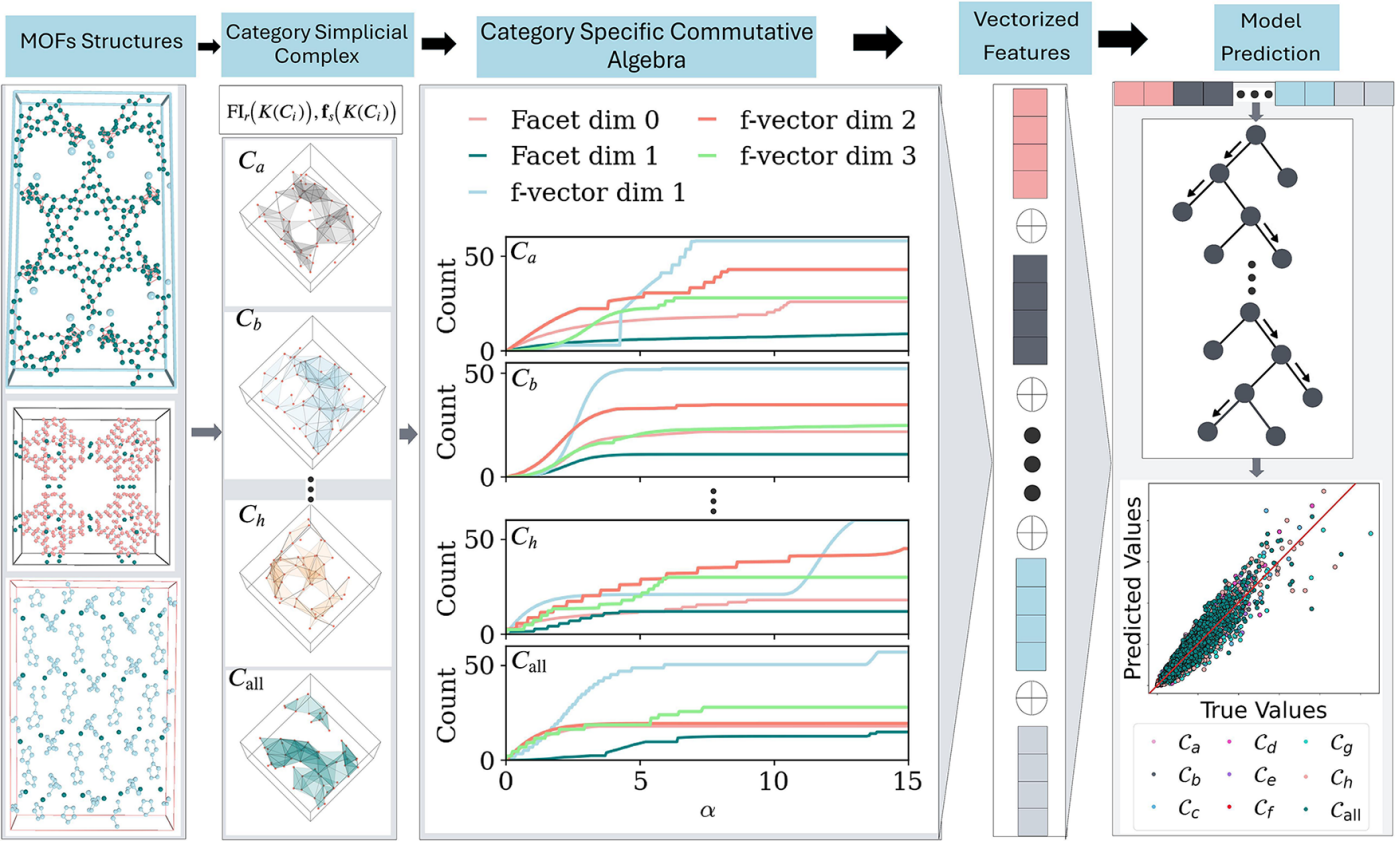
Category-Specific Commutative Algebra (CSCA) pipeline for MOF property prediction. (a) Starting point: each MOF structure is uniformly rescaled to a cubic supercell of side 64 Å. (b) Category simplicial complex construction: for each atom group *C*_*i*_ ∈ {*C*_*a*_,..., *C*_*h*_, *C*_all_}, atoms of that category are extracted and used to form the simplicial complex *K*(*C*_*i*_) using the α-complex filtration. (c) Algebraic curve generation: along the filtration parameter α, we compute two types of descriptors: facet interval curves *FI*_*r*_(*K*(*C*_*i*_);α) for dimensions *r* = 0, 1 (capturing connected components and loops) and *f*-vector curves *f*_*s*_(*K*(*C*_*i*_);α) for dimensions *s* = 1, 2, 3 (capturing counts of edges, triangles, and tetrahedra). (d) Feature vectorization: summary statistics of these curves are concatenated across all categories and dimensions into a unified feature representation. (e) Model prediction: the resulting feature vectors are used as input to a gradient boosting model, which predicts adsorption and transport properties of MOFs. This stepwise framework connects MOF structure to predictive machine learning via CSCA.

We build a simplicial representation for the atoms in each category *C*_*a*_,..., *C*_*h*_, *C*_all_. A *k*-simplex is the set determined by *k*+1 vertices. The 0-simplices are atoms or vertices, 1-simplices are bonds or edges, 2-simplices are triangles, and 3-simplices are tetrahedra. For each category *C*_*i*_ ∈ {*C*_*a*_,..., *C*_*h*_, *C*_all_}, we construct the α-complex on its atoms and take the downward closure to obtain the geometric simplicial complex *K*(*C*_*i*_); see Figure 6. With simplices included whenever their circumsphere radius is at most α, we build features from a radius filtration up to 12 Å. We record *f*-vector entries for edges, triangles, and tetrahedra and facet counts of dimensions 0 and 1 for all categories *C*_*a*_,..., *C*_*h*_, and *C*_all_. The *f*-vector describes how many bonds, surfaces, and 3-D cages exist at each radius α, while facet intervals measure the lifetimes of atoms and bonds before they are filled in by higher-dimensional structures. These curves are uniformly sampled throughout α and concatenated over *C*_*a*_,..., *C*_*h*_, and *C*_all_ to produce fixed-length vectors. Features designed based on these curves and their statistics are fed into gradient-boost decision tree (GBDT) models to build predictive models.

In our CSCA pipeline, algebraic descriptors are constructed directly from α-complex filtrations of the atomic coordinates, ensuring that all features arise from geometric proximity, coordination environments, and pore connectivity that are directly relevant to adsorption. As the filtration parameter α increases, simplices are created only when atoms become spatially accessible, linking algebraic persistence to physically meaningful length scales associated with adsorption sites and transport pathways. Category-specific decomposition localizes these algebraic constructions to chemically distinct environments, preserving the underlying physical heterogeneity of metal nodes and organic linkers, while persistence encodes multiscale algebraic invariants that track how connectivity evolves across length scales. Structural perturbations such as missing-linker defects, which are known to be tunable and to enhance gas adsorption in frameworks such as UiO-66,^7^ alter local coordination and pore accessibility; these changes naturally modify simplex formation and persistence and are therefore implicitly encoded in the resulting facet and *f*-vector descriptors without requiring explicit defect annotations.

Figure 2 shows the elemental distribution across the data set, which serves as the basis for defining our categories. For each category *C*_*a*_,..., *C*_*h*_, *C*_all_, Figure 2 reports the five most frequently occurring components ordering them according to how often they appear in the MOF data set. Carbon in *C*_*f*_, hydrogen in *C*_*e*_, and oxygen in *C*_*h*_ play significant roles in organic linkers and inorganic nodes, accounting for over 10,000 of MOFs. The relative abundance of nitrogen and phosphorus in *C*_*g*_ supports functional diversity in linkers. Zn and Cu appear in 2,358 and 2,022 MOFs, respectively, within *C*_*b*_, highlighting their importance as secondary building blocks. In contrast, elements in *C*_*d*_ and *C*_*c*_ (e.g., Cl: 481, Si: 119, B: 108 MOFs) occur far less frequently, indicating niche functional roles rather than backbone constituents. Overall, Figure 2 depicts the long tail contribution of rarer atoms that may have a significant impact on material characteristics as well as the dominance of core elements. This dominance is further corroborated by category-resolved feature importance and prediction accuracy analysis, which show that these frequently occurring elements contribute most consistently to the learned structure–property relationships.

**Figure 2:**
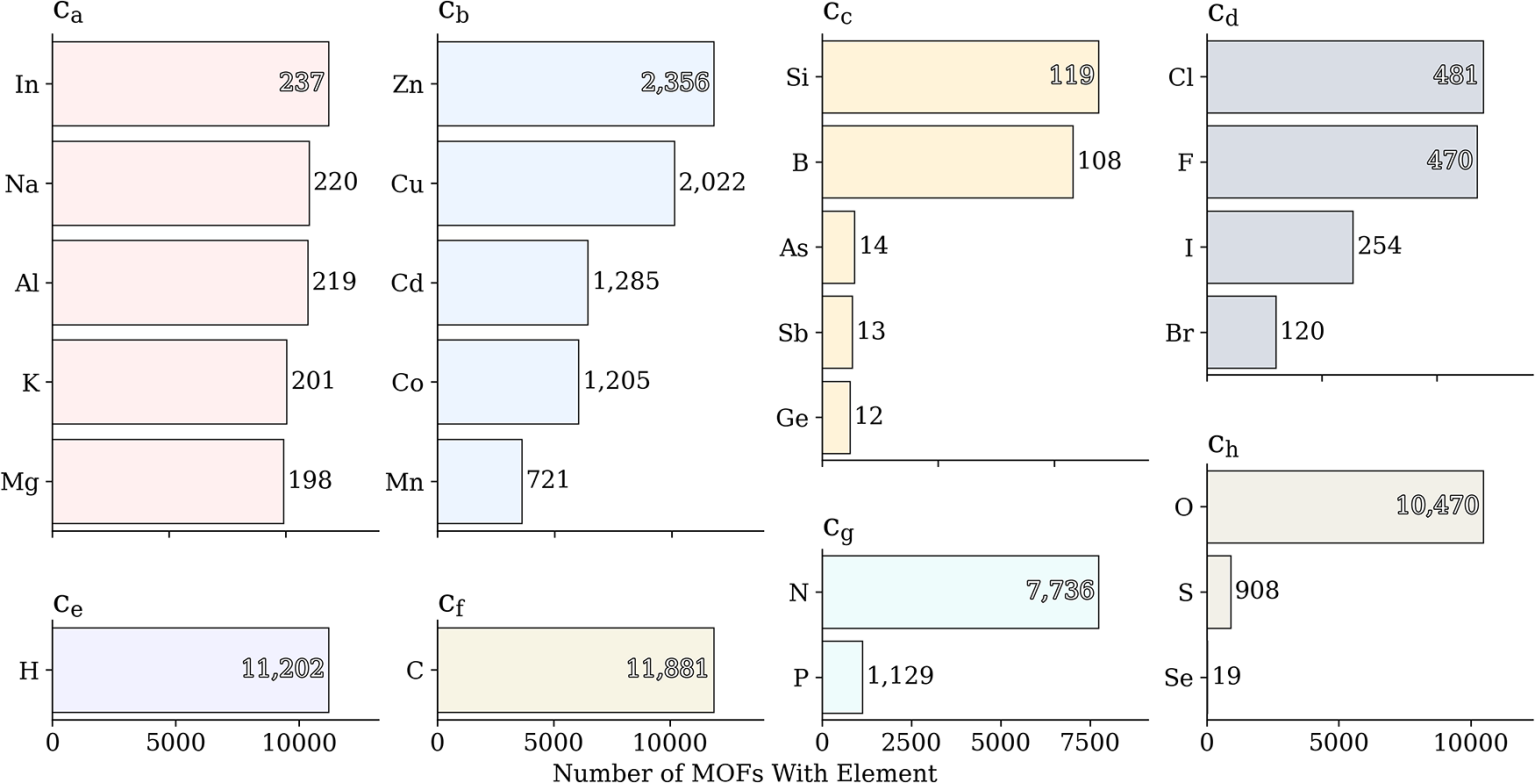
Top five most frequent elements within each compositional category *C*_*a*_,..., *C*_*h*_ in the CoRE MOF 2019 data set. Each bar represents the number of frameworks containing a given element. The distribution reveals prevalent elements in each chemical group, such as alkali and alkaline earth metals in *C*_*a*_, transition metals in *C*_*b*_, and light nonmetals (C, N, O, H) in *C*_*e*_,..., *C*_*h*_. These trends form the compositional backbone of the CSCA representation, linking chemical diversity to category-specific algebraic features used for adsorption-property prediction.

### 2.2 Data Set Overview

Our analysis centers on four property data sets: the Henry’s constants of oxygen and nitrogen, as well as their uptake capacities. The only property columns in each data set are O_2_ uptake (mol kg^–1^), N_2_ uptake (mol kg^–1^), Henry’s constant for O_2_, and Henry’s constant for N_2_. The element fraction ridgelines for these four property data set is shown in Figure 3. For each property in each data set, we compute the 25th and 75th percentiles and define two groups: Low for MOFs at the bottom 25% and High for MOFs at the top 25%. The middle 50% is not used in the ridge-line contrast. We then keep rows with a valid numeric property and convert the element count columns (H, C, N, O, metals, halogens) to the fraction of atoms in that MOF; this fraction is the number of atoms of that element divided by the total atom count, reported as a percentage. The Low or High partition is used exclusively for visualization to highlight compositional contrasts and does not affect model training or evaluation, which are performed on the full data set. Structural characteristics such as pore connectivity and multiscale framework geometry are captured by the α-complex–based facet and *f*-vector descriptors used in the model, ensuring that predictions are not driven by composition alone. Exact element occurrence counts and normalized fractions for each category are explicitly reported, and all compositional comparisons are performed using these normalized quantities rather than qualitative descriptors. We draw two ridge lines for each element: filled line for the High group and dashed line for the Low group.

**Figure 3:**
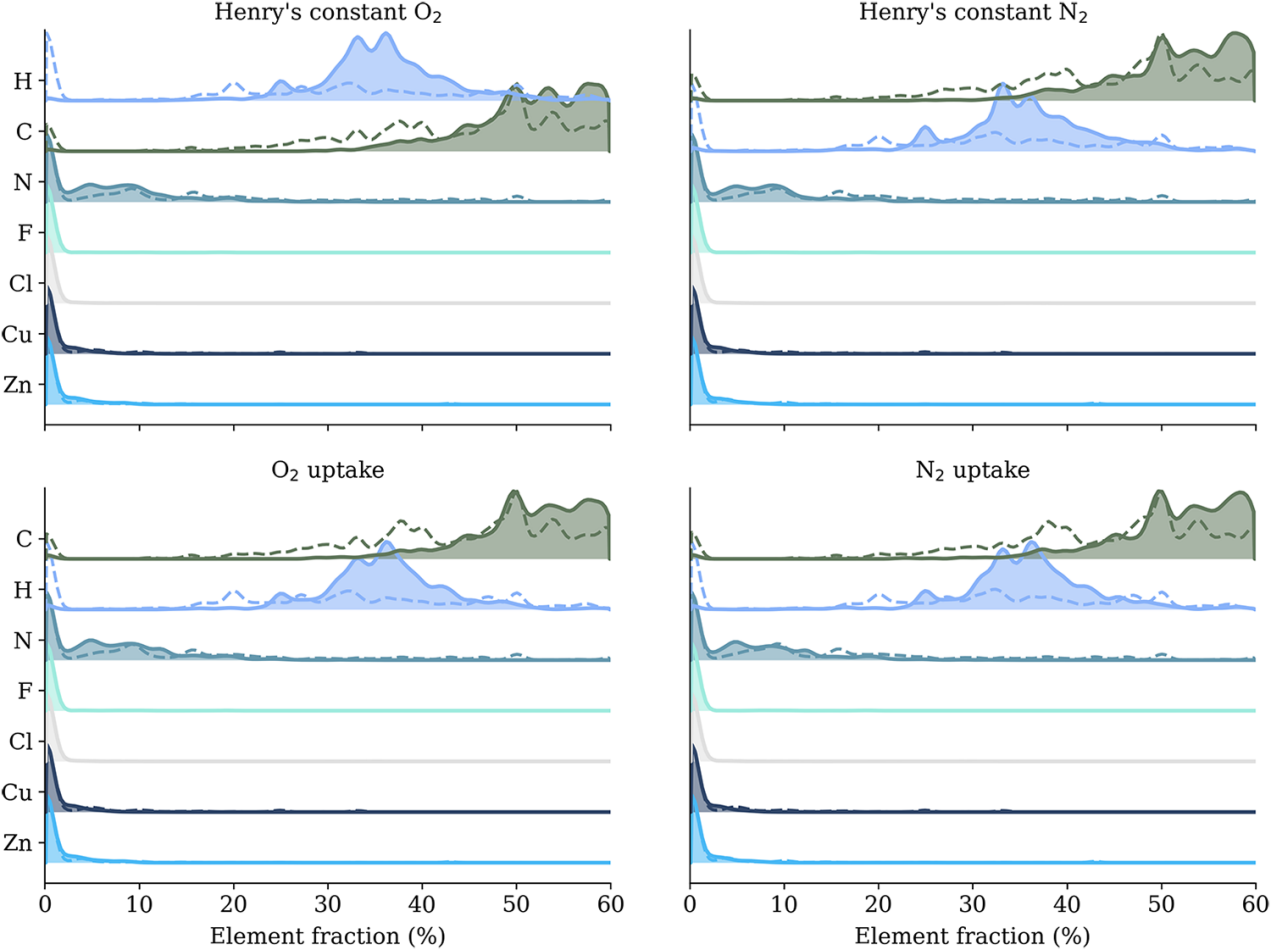
Element fraction ridgeline plots across four MOF property data sets. For each element, two distributions are shown: filled line = top 25% (MOFs with high property values) and dashed line = bottom 25% (MOFs with low property values). A rightward shift of the filled curve indicates enrichment in high-value MOFs. Observed trends: C and H are enriched; N shows mild enrichment; F, Cl, Cu, and Zn exhibit little or no shift.

A right shift of the filled curve means that the element is more abundant in MOFs with higher property values; a left shift means less; a strong overlap means little or no association. Quartiles provide two equal-sized groups, are robust to outliers, and avoid assuming a linear relationship. This lets us determine which atoms are associated with better performance for those properties. In all four panels, C and H shift right, N shows a small right shift, and F, Cl, Cu, and Zn stay near zero with substantial overlap.

### 2.3 Category-Specific Model Development in a Commutative Algebra Structure

We present category-specific topological representations for MOFs in this study. Table 5 outlines the classification of atoms into categories *C*_*a*_,..., *C*_*h*_ with additional category, *C*_all_, which contains all atoms. We create an α - complex and perform its filtration, capped at 12Å for every MOF and category (including *C*_all_). To describe higher order characteristics, we consider two sets of features. First, we compute *f*-vector components in dimension 1, 2, and 3 corresponding to the counts of edges, triangles, and tetrahedra. Second, we construct facet ideals in dimension 0 and 1, which capture algebraic relations among vertices and edges. Together, these features summarize both the combinatorial and algebraic aspects of the underlying structure. We also calculate summary statistics: mean, variance, minimum, and maximum of the corresponding filtration values for every category and simplex dimension to guarantee robustness and comparability. A single embedding vector that captures global organization together with category-specific commutative algebra invariants is produced by concatenating the facet ideal features, *f*-vectors features, and summary statistics across categories. This vector is then appropriate for further learning and analysis. Rather than replacing Betti numbers, the facet interval and *f*-vector curves used here complement classical topological invariants by tracking how simplices form and merge across scales, which is well suited for regression tasks in adsorption prediction.

### 2.4 Predicting MOF Properties from Category-Specific Commutative Algebra Features

In this study, four MOF properties are predicted to assess our category-specific commutative algebra (CSCA) model: uptake capacities for N_2_ and O_2_ (mol kg^–1^ Pa^–1^) and Henry’s constants for N_2_ and O_2_ (mol kg^–1^). Table 3 and Section 3.1 provide a detailed explanation of the data sets and their preparation.

The performance of our model on the four property data sets is summarized in Figure 4, and the performance of our model compared to other models is shown in Table 1. The prediction findings in Figure 4 show good agreement between the predicted and true values in the four data sets when training, validating and testing using a 80:10:10 split. For each data set, we average the results of 100 fits (10 random data splits, each trained with 10 independent seeds) of the model run with various seeds. By repeating random 80:10:10 splits over multiple runs, every MOF appears in the training, validation, and test sets across different splits, ensuring that all data points are explicitly exercised and contribute to the assessment of model stability. The accuracy and stability of the model are highlighted by the mean *R* ^2^ and MAE displayed in the upper left corner of each panel. Our category-specific commutative algebra model is benchmarked by comparing its performance to three large-scale baseline models: MOFTransformer, PMTransformer and Descriptor-based,^21,43,44^ where available. The category specific commutative algebra model utilizes a single universal set of hyperparameters presented in Table 5 across all data sets without validation-tuned settings, consistently outperforms descriptor baselines and competitive with or better than the transformer techniques as summarized in Table 1. The uniqueness of CSCA lies in the use of category-resolved commutative algebra descriptors rather than in the data-splitting protocol, which is intentionally aligned with prior descriptor-based studies: MOFTransformer, PMTransformer and Descriptor based^21,43,44^ to enable fair and direct performance comparison.

**Table 1:** A Comparison of CSCA Performance with Existing Models Across Multiple MOF Datasets

data set	method	*R* ^2^	MAE	RMSE
Henry’s constant N_2_	CSCA	0.78	5.30 × 10^–7^	7.74 × 10^–7^
	descriptor-based^44^	0.70		8.94 × 10^–7^
	MOFTransformer^21^			
	PMTransformer^43^			
Henry’s constant O_2_	CSCA	0.81	5.29 × 10^–7^	8.07 × 10^–7^
	descriptor-based^44^	0.74		9.60 × 10^–7^
	MOFTransformer^21^			
	PMTransformer^43^			
N_2_ uptake (mol kg^–1^)	CSCA	0.78	5.16 × 10^–2^	7.62 × 10^–2^
	descriptor-based^44^	0.71		8.62 × 10^–2^
	MOFTransformer^21^	0.78	7.10 × 10^–2^	
	PMTransformer^43^		6.90 × 10^–2^	
O_2_ uptake (mol kg^–1^)	CSCA	0.84	4.79 × 10^–2^	7.25 × 10^–2^
	descriptor-based^44^	0.74		9.28 × 10^–2^
	MOFTransformer^21^	0.83	5.10 × 10^–2^	
	PMTransformer^43^		5.30 × 10^–2^	

**Figure 4:**
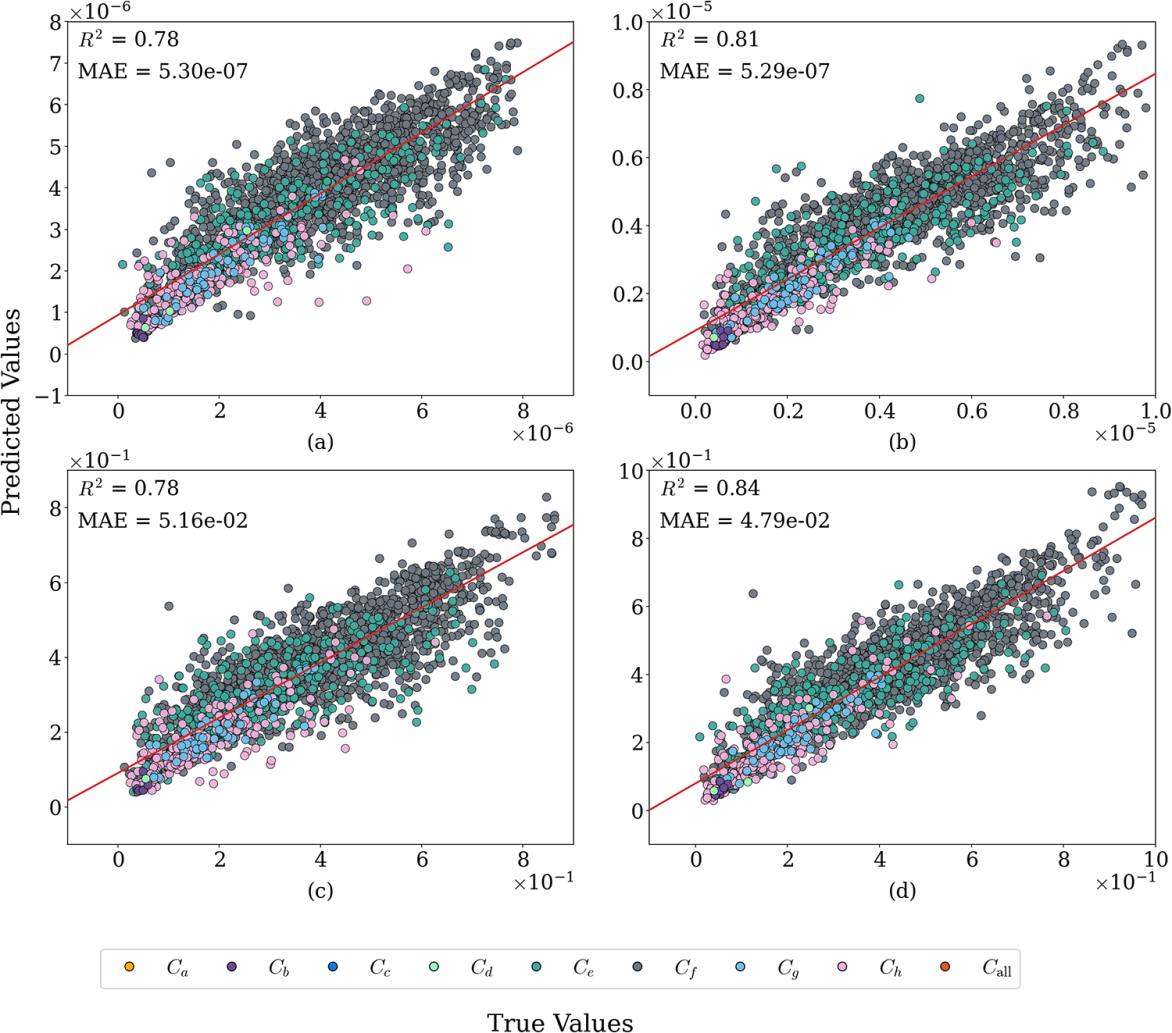
Predicted versus true values for the four property data sets: (a) Henry’s constants for N_2_; (b) Henry’s constants for O_2_; (c) uptake capacities for N_2_; and (d) uptake capacities for O_2_. Each point represents a single MOF sample, where the predicted value *ŷ*_*i*_ is obtained from the trained category-specific commutative algebra (CSCA) model using the corresponding feature vector of that sample. Points are colored according to their dominant atomic category (*C*_*a*_,..., *C*_*h*_ and *C*_all_), determined from atomic composition as defined in Table 4. All MOF samples are predicted by the same model; colors therefore indicate the primary chemical group to which each sample belongs rather than different feature types or separate models. Each panel reports two evaluation metrics in the upper-left corner: the mean absolute error (MAE) and the coefficient of determination (*R* ^2^), where *R* ^2^ measures the proportion of variance in the true values *y*_*i*_ explained by the predicted values *ŷ*_*i*_.

Color-coded categories demonstrate a significant class imbalance, with *C*_*f*_ accounting for the vast majority of samples, as seen in Figure 4. In all four data sets, there is no discernible class division; instead, the categories merge into a single overlapping diagonal band. Although minority categories emerge as thinner overlays that reveal somewhat larger dispersion, particularly in mid to high ranges, *C*_*f*_ generally sustains the dense core along the regression line. Similar scaling behavior is shown at the extremes, where all categories taper but stay in line with the diagonal. The abundance of *C*_*f*_ dominates the global metrics *R* ^2^ and MAE, but there is no indication of category-specific bias because every category exhibits the same increasing trend. However, there is a slight variation in accuracy; *C*_*g*_ and *C*_*b*_ cluster more closely around the regression line, whereas *C*_*h*_ and *C*_*e*_ shows higher dispersion at lower to mid values. Overall, this suggests that the high global performance represents consistent predicted accuracy across chemistry rather than being only a result of the majority class. The overlap of predictions across categories reflects a shared structure–property relationship rather than a loss of category-specific information. Although different categories map to similar target values, CSCA retains interpretability through distinct algebraic feature contributions and category-resolved accuracy. Thus, category-specific insight arises from the descriptors themselves, not from visual separation in predicted-versus-true space.

To assess whether the apparent overlap of predictions across chemical categories reflects category-dependent bias, we report category-resolved prediction accuracy for all four adsorption properties. Although the predicted-versus-true scatter plots show substantial overlap among categories, Table 2 demonstrates that mean absolute error and *R* ^2^ remain comparable across all elemental groups. No systematic degradation is observed for underrepresented categories, indicating that the diagonal overlap reflects a shared structure–property relationship rather than dominance by the majority class.

**Table 2:** Presence-Based Category-Resolved Test Performance for Four Adsorption Properties^a^

	Henry N_**2**_		Henry O_**2**_		N_2_ uptake		O_2_ uptake	
category	MAE	*R* ^**2**^	MAE	*R* ^**2**^	MAE	*R* ^2^	MAE	*R* ^2^
*C*_*a*_	6.61 × 10^–7^ ± 1.25 × 10^–7^	0.67 ± 0.14	7.07 × 10^–7^ ± 2.42 × 10^–7^	0.72 ± 0.19	0.07 ± 0.01	0.56 ± 0.19	0.06 ± 0.01	0.76 ± 0.08
*C*_*b*_	5.11 × 10^–7^ ± 2.40 × 10^–8^	0.78 ± 0.03	5.47 × 10^–7^ ± 2.04 × 10^–8^	0.82 ± 0.02	0.05 ± 0.00	0.76 ± 0.03	0.05 ± 0.00	0.84 ± 0.03
*C*_*c*_	6.94 × 10^–7^ ± 8.56 × 10^–8^	0.65 ± 0.11	7.03 × 10^–7^ ± 1.16 × 10^–7^	0.73 ± 0.09	0.06 ± 0.01	0.64 ± 0.10	0.06 ± 0.01	0.78 ± 0.05
*C*_*d*_	4.85 × 10^–7^ ± 4.99 × 10^–8^	0.73 ± 0.09	5.33 × 10^–7^ ± 5.10 × 10^–8^	0.80 ± 0.07	0.05 ± 0.00	0.78 ± 0.06	0.05 ± 0.00	0.84 ± 0.05
*C*_*e*_	5.43 × 10^–7^ ± 2.18 × 10^–8^	0.74 ± 0.03	5.60 × 10^–7^ ± 2.03 × 10^–8^	0.81 ± 0.02	0.05 ± 0.00	0.76 ± 0.03	0.05 ± 0.00	0.84 ± 0.03
*C*_*f*_	5.30 × 10^–7^ ± 2.15 × 10^–8^	0.78 ± 0.03	5.29 × 10^–7^ ± 2.04 × 10^–8^	0.83 ± 0.02	0.05 ± 0.00	0.77 ± 0.03	0.05 ± 0.00	0.86 ± 0.02
*C*_*g*_	5.40 × 10^–7^ ± 2.82 × 10^–8^	0.76 ± 0.04	5.63 × 10^–7^ ± 2.68 × 10^–8^	0.81 ± 0.03	0.05 ± 0.00	0.77 ± 0.04	0.05 ± 0.00	0.85 ± 0.03
*C*_*h*_	5.34 × 10^–7^ ± 2.15 × 10^–8^	0.77 ± 0.03	5.56 × 10^–7^ ± 2.05 × 10^–8^	0.82 ± 0.02	0.05 ± 0.00	0.76 ± 0.03	0.05 ± 0.00	0.84 ± 0.03

a Mean ± standard deviation are reported over 10 random data splits; within each split, predictions are averaged over 10 independent model seeds.

Although CSCA generates a high-dimensional descriptor set (5598 features per MOF), predictive performance is driven by a small, consistent subset of descriptors. Feature-importance profiles in the Supporting Information and discussions in 2.6 indicate that facet dimension 1 and *f*-vector dimensions 1 and 2 dominate across properties and scales, indicating meaningful multiscale structure encoding rather than redundancy among correlated features.

Table S2 in the Supporting Information summarizes the predictive performance of the CSCA model on four adsorption-related properties. For each target, results are reported as the mean and standard deviation over 10 independent 80:10:10 train-validation-test splits, with predictions in each split averaged across 10 Gradient Boosting models trained with different random seeds. Model performance is evaluated using *R* ^2^, mean absolute error (MAE), and root mean squared error (RMSE), where higher *R* ^2^ and lower error values indicate improved predictive accuracy.

### 2.5 Feature Construction

We partition atoms into element groups *C*_*a*_,..., *C*_*h*_ and *C*_all_. For each category *C*_*i*_ ∈ {*C*_*a*_,..., *C*_*h*_, *C*_all_}, we restrict the previously built α-complex to the subcomplex *K*(*C*_*i*_). With a radius filtration α ∈ [0, 12] Å at resolution Δ*α* = 0.1 Å (121 thresholds), we record five curves per category (facet-count in dimensions 0 and 1; *f*-vector in dimensions 1,2,3), yielding 5 × 121 = 605 entries, together with 16 facet summary statistics derived from persistence intervals in dimensions 0 and 1. For each dimension, we record the count of intervals, total persistence, maximum, mean, median, and the lower and upper (25%) quartiles of lifetimes. Thus, each category contributes 605 + 16 + 1 = 622 features, and across nine categories (*C*_0_,..., *C*_7_, *C*_all_) the total is 9 × 622 = 5,598 features per MOF. The discretized curves are concatenated across all categories to form a single category aware commutative algebra descriptor per MOF. A single 2-D t-SNE of these descriptors is shown in Figure 5a. The figure places MOFs into coherent clusters; we mark property extremes for the four targets with triangles and small circles representing maximum and minimum values of the four properties, respectively. The separation of these highlights indicates that the commutative algebra features already encode the key structure property variations relevant to adsorption and transport. This plot illustrates that MOFs with extreme adsorption properties do not scatter randomly but instead fall into recognizable regions of the feature space.

**Figure 5:**
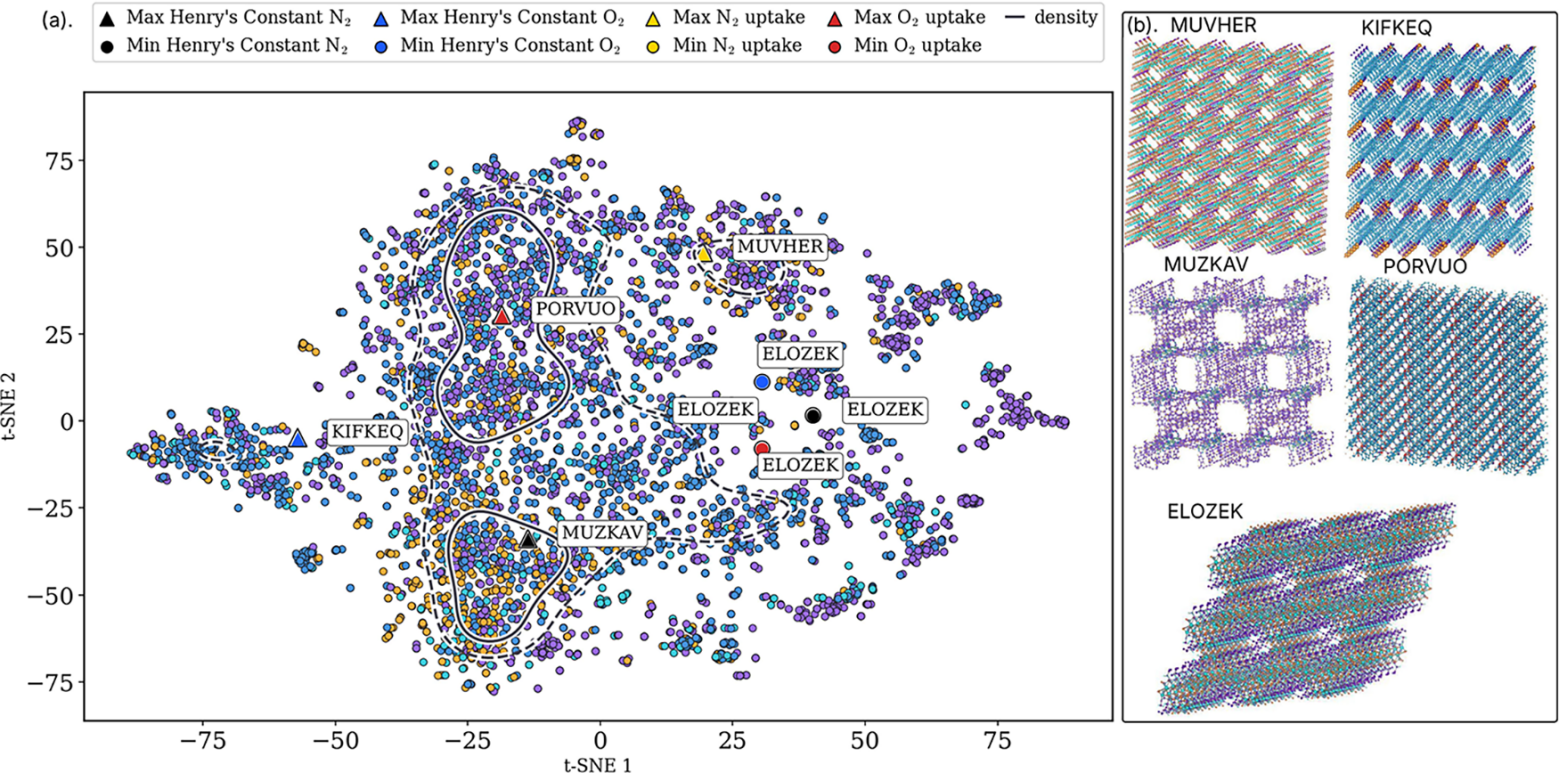
(a) t-SNE of category-specific commutative algebra features. Background points show the data set; colored markers highlight the maxima and minima for the four properties. We computed two-dimensional t-SNE embedding with perplexity equal to 35, number of iterations equal to 1500, and random seed equal to 42 on our standardized α facet and *f*-vector features. Solid contours indicate kernel density level sets at the 85 and 95% quartiles. (b) Representative MOFs at property extrema. The selected structures illustrate the diversity of network geometries, pore architectures, and elemental compositions associated with extreme adsorption behaviors. Specifically, MUZKAV exhibits the highest Henry’s constant for N_2_ (7.89 × 10^–6^ mol kg^–1^ Pa^–1^), while KIFKEQ attains the maximum Henry’s constant for O_2_ (9.79 × 10^–6^ mol kg^–1^ Pa^–1^). ELOZEK shows the lowest Henry’s constants for both N_2_ and O_2_ (8.86 × 10^–8^ mol kg^–1^ Pa^–1^) and also demonstrates the minimum uptake capacities for N_2_ and O_2_ (0.0085 mol kg^–1^ and 0.0086 mol kg^–1^, respectively). In contrast, MUVHER and PORVUO exhibit the highest uptake capacities for N_2_ (0.98 mol kg^–1^) and O_2_ (1.11 mol kg^–1^), respectively. These structures highlight the range of adsorption responses captured by the CSCA framework.

### 2.6 Feature Importance

Facet dimension 1 and the *f*-vector dimensions 1 and 2 provide the dominating signal across categories *C*_*a*_,..., *C*_*h*_ and *C*_all_, see Section S1. The majority of the predictive power is enabled by local to medium connectivity, as indicated by the concentration of peaks at small to intermediate filtration scales α ≤ 5Å. Carbon in *C*_*f*_ and hydrogen in *C*_*e*_ rise early in facet dimension 1 and *f*-vector dimensions 1 and 2; oxygen, sulfur, and selenium in *C*_*h*_ give broader profiles; metals in *C*_*b*_ exhibit sharp, confined responses, particularly in *f*-vector dimension 2; and *f*-vector dimension 3 only appears as selective, lower magnitude peaks (for instance, in *C*_*f*_ and *C*_all_). These patterns hold for both uptakes and Henry’s constants (Figures S2–S4).

Across element based categories *C*_*a*_ to *C*_*h*_ and the pooled set *C*_all_, the most informative descriptor families are facet dimension 1 and *f*-vector dimensions 1 and 2, which show dominant peaks at small to intermediate filtration scales (α ≤ 5 Å) in the feature-importance profiles (Figures S2–S4). The strongest contributions arise from *C*_*a*_ (alkali and alkaline-earth metals), *C*_*g*_ (nitrogen–phosphorus), and *C*_*h*_ (oxygen–sulfur–selenium), indicating that short-range and medium-range polar or charged environments govern MOF selectivity toward N_2_ and O_2_, while framework carbons and nonpolar linkers (*C*_*f*_) primarily shape geometric confinement rather than selective adsorption.

In *C*_*a*_, facet dimension 1 and *f*-vector dimension 2 are significant for all four properties, emphasizing the importance of local carbon framework geometry across tasks. *C*_*b*_ exhibits abrupt, narrow peaks in *f*-vector dimension 2, particularly for Henry’s constants N_2_ and O_2_ in Figures S1 and S2, respectively, indicating hydrogen’s effect at highly confined structural scales. *C*_*c*_ and *C*_*d*_ provide larger, multiscale contributions, as seen in Figures S3 and S4 for N_2_ and O_2_ uptakes respectively, demonstrating that metal-centered connectivity and O-linked nodes impact uptake via longer-range structural organization. Intermediate categories *C*_*e*_ to *C*_*g*_ often include substantial peaks in facet dimension 1 and *f*-vector dimension 1, which serve as supportive contributors. Finally, *C*_*h*_ commonly exhibits larger significance profiles, most notably for O_2_ uptake in Figure S4, indicating oxygen’s continued engagement in linker node connection, which influences overall transport. Figures S1 and S2 for Henry’s constants N_2_ and O_2_ respectively show clearly concentrated significance peaks at certain α values, demonstrating sensitivity to micropore environments and first contact adsorption sites. N_2_ and O_2_ Uptake in Figures S3 and S4 respectively show a more dispersed relevance across α and categories, indicating the necessity to reflect multiscale connectivity and accessible volume that influence loading beyond the initial adsorption event. Despite these variations, the dominance of facet dimension 1 and *f*-vector dimensions 1 and 2 is a continuous cross-property characteristic. These feature analysis demonstrates that descriptor families encoding local-to-medium scale morphology facet dimension 1, *f*-vector dimension 1 and 2 are most predictive, while large α features contribute comparatively little. Different element categories specialize: carbons and hydrogens dominate localized effects, while metals and oxygen extend predictivity over broader scales, especially for uptake. These data give clear direction for descriptor selection, prioritize facet dimension 1 and *f*-vector dimensions 1 and 2, and imply that models aiming uptake should focus on multiscale features covering various α ranges. From a physical standpoint, the dominant CSCA descriptors admit direct chemical interpretation. Facet intervals in dimension 1 quantify the persistence of bonded connections before higher-dimensional filling, reflecting the stability of local coordination environments that govern initial adsorption. Likewise, *f*-vector components in dimensions 1 and 2 capture edge and triangular motifs associated with pore throat connectivity and accessible surface geometry. The concentration of importance at small to intermediate filtration scales (α ≤ 5Å) therefore links predictive signals to chemically intuitive micropore-scale environments rather than abstract global connectivity, distinguishing CSCA interpretability from implicit representations learned by deep learning models. In contrast to deep learning models, where learned representations are implicit and distributed, each CSCA feature has a fixed algebraic definition and can be traced back to a specific geometric and chemical motif within the framework.

Category partitioning and persistent commutative algebra play complementary roles in the CSCA framework. Category partitioning endows algebraic descriptors with chemically and physically distinct traits, such as metal nodes, linker backbones, and heteroatom groups, thereby preserving the underlying physics of MOF interactions. In contrast, persistent commutative algebra constructions, including α-complex filtrations, facet-interval statistics, and multiscale *f*-vector curves, encode higher-order connectivity and the evolution of structural organization across length scales. The predictive gains of CSCA arise from the integration of these two components: category partitioning alone lacks multiscale robustness, while persistence alone obscures chemical specificity. Their combination enables accurate and interpretable modeling of adsorption properties.

## 3 Methods

### 3.1 Data Sets

The data set comprised of structures from the CoRE MOF 2019 database, which is widely used in comparable work.^25,45^ We focus on adsorption properties: Henry coefficients for O_2_ and N_2_, selectivity derived from these coefficients, and uptake capacities. We use the filtering method of Orhan et al.,^44^ removing entries that exceeded property-specific upper bounds and excluding within sigma outliers. As a result, the data set obtained was more consistent and representative of the property space of interest. Additionally, upper limit thresholding was employed to reduce bias and boost the robustness of the predictive models.^44^ After cleaning and preprocessing, the data set was split into training, validation, and test groups using an 80:10:10 ratio as in.^25^ Table 3 provides a summary of the final data set used in this study, along with the properties considered, the corresponding number of samples and the randomized splitting approach employed for model development.

**Table 3:** Dataset Statistics for O_2_ and N_2_ Adsorption and Transport Characteristics in MOFs^25^

property	units	samples	split	ratio
Henry’s constant of N_2_	mol kg^–1^ Pa^–1^	4744	random	8:1:1
Henry’s constant of O_2_	mol kg^–1^ Pa^–1^	5036	random	8:1:1
N_2_ uptake	mol kg^–1^	5132	random	8:1:1
O_2_ uptake	mol kg^–1^	5241	random	8:1:1

### 3.2 Simplicial Complex

Let *V* = {1,..., *n*} be a finite set of vertices. A simplex is any finite subset σ ⊆ *V*. A simplicial complex on *V* is a family *K* ⊆ 2^*V*^ satisfying the closure condition

1 σ∈K,τ⊆σ⇒τ∈K

so every face of a simplex is also in *K*. We also require every vertex to appear: {*v*} ∈ *K* for all *v* ∈ *V*.

Elements of *K* are called faces. A facet is a face that is not contained in any larger face, and the set of all facets is

2 F(K)={τ∈K:∃σ∈Ksuch thatτ⊆σ}

The dimension of a face is dimσ = |σ| – 1, and the dimension of the complex is

3 $$\dim K=\max_{\sigma \in K}\dim\sigma$$

For a vertex subset *W* ⊆ *V*, the induced subcomplex is

4 $$K_{W}=\left\{\sigma \in K:\sigma \subseteq W\right\}$$

### 3.3 Stanley-Reisner Theory

Let k be a field and let

5 $$S=k\left[x_{1},...,x_{n}\right],,\deg{\;x}_{i}=1$$

be the polynomial ring whose variables *x*_*i*_ correspond to the vertices *i* ∈ *V*. To each face σ ⊆ *V* associate the squarefree monomial

6 $$x^{\sigma }=\prod_{i\in \sigma }x_{i},,x^{⌀}=1$$

so that each subset of vertices is encoded by a distinct monomial. The Stanley–Reisner ideal of *K* records the nonfaces

7 $$I\left(K\right)=\left\langle x^{\sigma }:\sigma \subseteq V,\sigma \notin K\right\rangle \subseteq S$$

and the corresponding Stanley–Reisner ring is

8 k[K]=S/I(K)

Thus, monomials corresponding to actual faces remain nonzero in k[K] , while those corresponding to nonfaces vanish. For each facet τ∈F(K) , the facet prime is

9 $$P_{\tau }=\left(x_{i}:i\notin \tau \right)\subset S$$

These are the minimal prime ideals of *I*(*K*), and one has the irredundant decomposition

10 $$I\left(K\right)=\underset{\tau \in F\left(K\right)}{\cap }P_{\tau }$$

A minimal graded free resolution of k[K] over the polynomial ring $S=k\left[x_{1},...,x_{n}\right]$ is an exact sequence of free *S*-modules

11 $$\cdot \cdot \cdot \to \underset{j}{⊕}S{\left(-j\right)}^{\beta_{i,j}}\to \cdot \cdot \cdot \to \underset{j}{⊕}S{\left(-j\right)}^{\beta_{0,j}}\to k\left[K\right]\to 0$$

Each term *S*(−*j*) represents a graded shift of *S* by degree *j*, and the integers β_*i*,*j*_ are the graded Betti numbers that record how many generators or relations occur at each homological degree *i* and internal degree *j*. They can be computed algebraically as

12 $$\beta_{i,j}=\dim_{k}{\left({\mathrm{Tor}}_{i}^{S}\left(k\left[K\right],k\right)\right)}_{j}$$

where ${\mathrm{Tor}}_{i}^{S}\left(k\left[K\right],k\right)$ measures the dependencies among generators at level *i*, and the subscript *j* extracts the component of total degree *j*.

Hochster’s formula connects them to simplicial homology

13 $$\beta_{i,i+j}\left(k\left[K\right]\right)=\sum_{\begin{array}{l} W\subseteq V\\ \left|W\right|=i+j \end{array}}\dim_{k}{\mathrm{H̃}}_{j-1}\left(K_{W};k\right)$$

Here *K*_*W*_ is the subcomplex induced by vertex subset *W*, and ${\mathrm{H̃}}_{j-1}\left(K_{W};k\right)$ denotes the reduced homology group in dimension *j*–1 with coefficients in k . Its dimension counts the number of independent (*j*–1)-dimensional holes in *K*_*W*_.

If dim *K* = *d*–1, the Hilbert series of k[K] has the form

14 $$H_{K}\left(t\right)=\frac{h_{0}+h_{1}t+\cdot \cdot \cdot +h_{d}t^{d}}{{\left(1-t\right)}^{d}}$$

The coefficients *h*(*K*) = (*h*_0_,..., *h*_*d*_) define the *h*-vector. The *f*-vector counts faces by dimension

15 $$f\left(K\right)=\left(f_{-1},f_{0},...,f_{d-1}\right),,f_{i}=\neq \left\{\sigma \in K:\dim\sigma =i\right\},,f_{-1}=1$$

They are related by

16 $$\sum_{j=0}^{d}h_{j}t^{j}=\sum_{j=0}^{d}f_{j-1}t^{j}{\left(1-t\right)}^{d-j}$$

### 3.4 Filtration

The Čech complex, the α-complex, and the Vietoris Rips complex^46–48^ are the three filtrations that are often employed in topological data analysis. All three yield nested sequences of simplicial complexes that enlarge as the scale parameter increases. We use the α-complex in this study, which is geometrically appropriate for atomic coordinates in periodic MOFs. A simplex (edge, triangle, or tetrahedron) is added once the radius of its lowest empty circumsphere is at most α. It is built using the Delaunay triangulation. Figure 6 shows an illustration of a filtration. As α grows, the complexes expand by subset inclusion

17 $$K^{\leq \alpha }\subseteq K^{\leq \alpha \prime }\;\left(\alpha \leq \alpha^{\prime }\right),,\underset{\alpha \geq 0}{\cup }K^{\leq \alpha }=K$$

**Figure 6:**
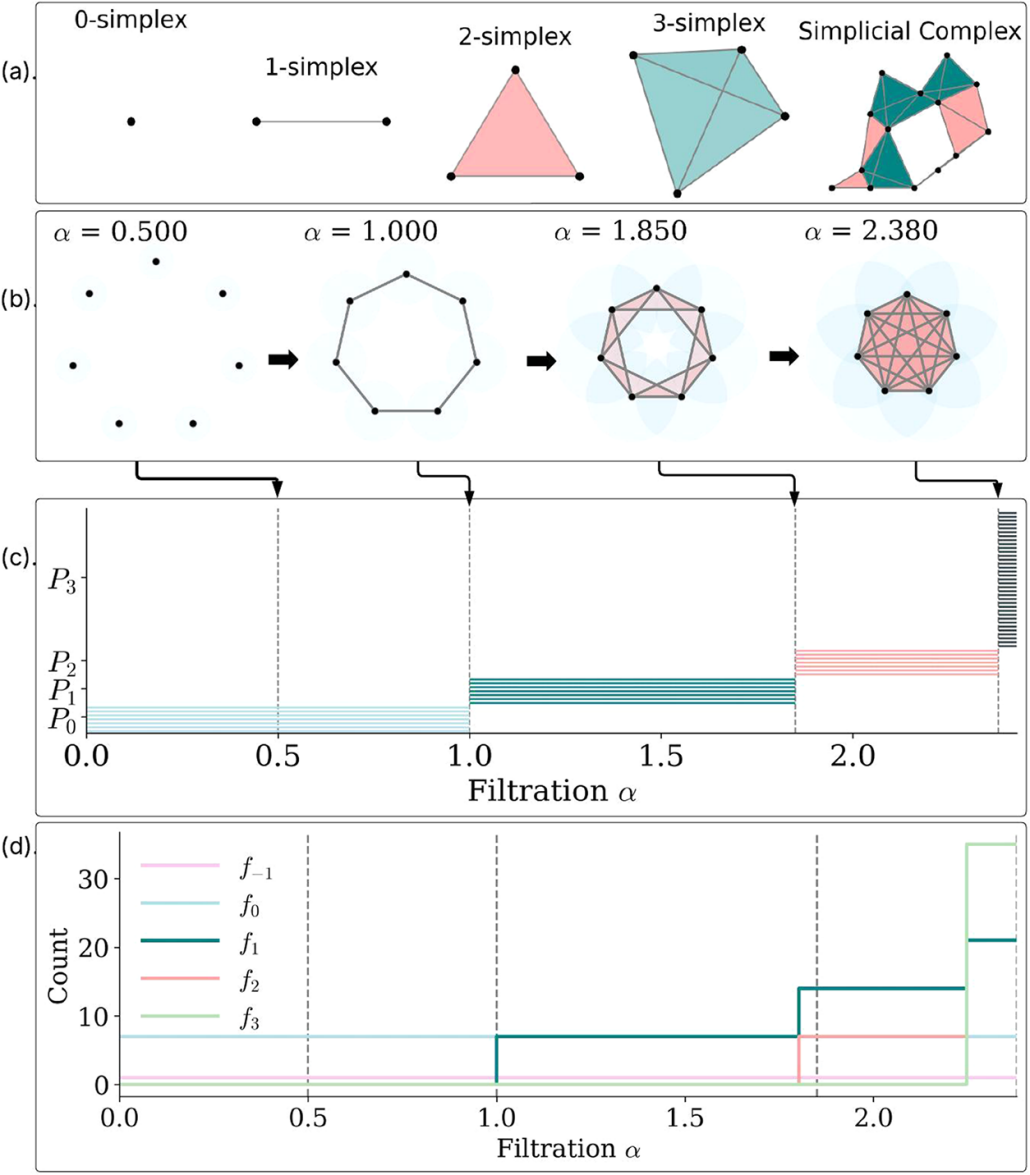
Simplicial filtration and summary statistics. (a) The representation of simplices in dimensions 0,1,2 and 3: (b) filtration of an α-complex on a 2 dimension heptagon random point cloud, α-complex snapshots for α ∈ {0.50, 1.00, 1.85, 2.38}; previously present simplices are faded, newly added ones are highlighted; translucent α-balls show the scale: (c) summary plots: facet barcodes by dimension (*P*_0_, *P*_2_, *P*_3_): (d) *f*-vector curves *f*_*k*_(α) for *k* = – 1, 0, 1, 2, 3.

The birth of a face σ is the smallest scale where it appears

18 $$b\left(\sigma \right)=\inf\left\{\alpha :\sigma \in K^{\leq \alpha }\right\}$$

The facet intervals in dimension *i* record the persistence of facets of dimension *i*

19 $${\mathrm{FI}}^{\left(i\right)}\left(K\right)=\left\{\left[\mathrm{b̂}\left(\tau \right),\mathrm{d̂}\left(\tau \right)\right):\tau \;\mathrm{an}\;i-\mathrm{facet}\;\mathrm{at}\;\mathrm{some}\;\mathrm{scale}\right\}$$

Here *b̂*(τ) and *d̂*(τ) denote the birth and death scales of the facet τ, respectively. The birth scale *b̂*(τ) is the smallest value of α for which τ appears in the filtration *K* ^≤α^, while the death scale *d̂*(τ) is the smallest value of α at which τ ceases to be a facet, that is, when it becomes a face of a higher-dimensional simplex. Therefore, each interval [*b̂*(τ), *d̂*(τ)] represents how long the facet τ persists during the filtration.

The combinatorial growth of the complex is summarized by the *f*-vectors

20 $$f_{i}\left(\alpha \right)=\#\left\{\sigma \in K^{\leq \alpha }:\dim\sigma =i\right\},,i\geq 0$$

which track how many vertices, edges, triangles, and tetrahedra are present at each scale.

### 3.5 Category-Restricted Complexes

Let G={a,b,c,d,e,f,g,h,all} denote the element categories. For each i∈G , define

21 $$K_{i}=K\left(C_{i}\right)$$

the subcomplex induced on vertices of category *C*_*i*_ (*K*_all_ is the full complex). Each *K*_*i*_ inherits the α-complex filtration (17), so that

22 $$K_{i}^{\leq \alpha }\subseteq K_{i}^{\leq \alpha \prime }\left(\alpha \leq \alpha^{\prime }\right)$$

For every category and dimension, we record

23 $$F_{j}^{\left(i\right)}\left(\alpha \right)=\left\{\sigma \in K_{i}^{\leq \alpha }:\dim\sigma =j\right\}$$

24 $$f_{j}^{\left(i\right)}\left(\alpha \right)=\left|F_{j}^{\left(i\right)}\left(\alpha \right)\right|$$

25 $${\mathrm{Facet}}_{j}^{\left(i\right)}\left(\alpha \right)=\left\{\tau \in K_{i}^{\leq \alpha }:\tau \;a\;j-\mathrm{facet}\right\}$$

Facet intervals for category *i* are

26 $${\mathrm{FI}}^{\left(j\right)}\left(K_{i}\right)=\left\{\left[\mathrm{b̂}\left(\tau \right),\mathrm{d̂}\left(\tau \right)\right):\tau \in {\mathrm{Facet}}_{j}^{\left(i\right)}\left(\alpha \right)\right\}$$

The *f*-vector curves are

27 $$f_{1,2,3}^{\left(i\right)}\left(\alpha \right)=\left(f_{1}^{\left(i\right)}\left(\alpha \right),f_{2}^{\left(i\right)}\left(\alpha \right),f_{3}^{\left(i\right)}\left(\alpha \right)\right)$$

which describe the evolution of edges, triangles, and tetrahedra within each element category.

### 3.6 Feature Vector

The final feature vector concatenates, over all category features, statistical summaries of facet intervals ${\mathrm{FI}}^{\left(j\right)}\left(K_{i}\right)$ for *j* = 0,1, together with sampled values of the *f*-vector curves (27). This combines persistence based information from facets with combinatorial counts from *f*-vectors, providing a joint representation of how structural connectivity evolves across scales and categories.

### 3.7 Category-Specific Commutative Algebra Embedding

Graph-based models are frequently used in traditional representations of MOF topology, in which bonds are represented as edges and atoms as nodes.^25^ This model is useful for capturing pairwise interactions, but it is unable to represent higher-order structural connections that are essential for stability, adsorption, and diffusion.^25^ A particular type of simplicial complex that offers a more sophisticated algebraic topological framework is α - complexes, which we use to get around this restriction. Due to their derivation from the Delaunay triangulation, α - complexes maintain geometric accuracy while drastically lowering duplication, in contrast to general Vietoris Rips complexes.^49^ Our method first scales MOF structures into a homogeneous supercell of 64Å × 64 Å × 64 Å, ensuring uniformity across all data sets. Following that, atoms are categorized into several groups based on their structural properties and chemical similarities, as shown in Table 4. In order to summarize higher order simplices, we compute *f*-vectors in dimensions 1, 2, and 3 and extract facet intervals of dimensions 0 and 1 from these complexes. We obtain summary statistics of the corresponding filtration values, including the mean, variance, minimum, and maximum, for each category and simplex dimension in order to make the representation robust and comparable. The analysis is limited to physically relevant scales by truncating the filtration at a maximum radius α_max_ = 12 Å. Features for machine learning and analysis are obtained by concatenating the interval features, *f*-vectors, and summary statistics over all categories.

**Table 4:** Element Categories $C_{a},...,C_{h},C_{\mathrm{all}}$ Used for Category-Specific Topological Modeling of MOFs

element category	notation	elements included
alkali metals, alkaline earth metals, and post-transition metals	*C*_*a*_	Li, Na, K, Rb, Cs; Be, Mg, Ca, Sr, Ba; Al, Ga, In, Sn, Pb, Bi
transition metals, lanthanides, actinides	$C_{b}$	Sc, Ti, V, Cr, Mn, Fe, Co, Ni, Cu, Zn; Y, Zr, Nb, Mo, Ru, Rh, Pd, Ag, Cd; Hf, Ta, W, Re, Os, Ir, Pt, Au, Hg; La, Ce, Pr, Nd, Sm, Eu, Gd, Tb, Dy, Ho, Er, Tm, Yb, Lu; Th, U
metalloids	*C*_*c*_	B, Si, Ge, As, Sb, Te
halogens	*C*_*d*_	F, Cl, Br, I
hydrogen	*C*_*e*_	H
carbon	*C*_*f*_	C
nitrogen, phosphorus	*C*_*g*_	N, P
oxygen, sulfur, selenium	*C*_*h*_	O, S, Se
all elements (union of *C*_*a*_,..., *C*_*h*_)	*C*_all_	all of the above

Figure 6 shows a filtration built by a simple ball rule: at each scale α, we draw a ball of radius α around every point. A vertex is present for all α; an edge appears when the two balls intersect; a triangle appears when all three pairwise edges are present (equivalently, the three balls have a common intersection); and a tetrahedron appears when all six edges among four vertices are present. Thus, at α = 0.50 only vertices are present; at α = 1.00 the outer-ring edges appear; at α = 1.85 the first triangles appear; and by α = 2.38 tetrahedra also appear. For each facet σ we define its birth *b*(σ) as the smallest α at which it first exists under this rule, and its death *d*(σ) as the smallest α at which it first becomes a face of a higher-dimensional facet. The barcode in panel (b) of Figure 6 draws one [*b*(σ), *d*(σ)] per facet, grouped by dimension $P_{k}$ , where $P_{0}$ denotes vertices (dimension 0), $P_{1}$ edges (dimension 1), $P_{2}$ triangles (dimension 2), and $P_{3}$ tetrahedra (dimension 3). Accordingly, panel (b) shows 7 vertex bars in [0, 1.00], 7 edge bars in [1.00, 1.85], 7 triangle bars in [1.85, 2.38], and 35 tetrahedron bars at [2.38, ∞]. Panel (c) tracks how the number of active facets evolves with α which is represented by the *f*-vector curves *f*_*k*_(α) where *k* is the dimension. We plot *f*_*k*_(α) = # {k facets alive at α}, so curves step up at births, levels when the facets persists and step down at deaths. At the marked scales, the values (in order (*f*_–1_,*f*_0_,*f*_2_,*f*_3_)) are 0.50:(1, 7, 0, 0), 1.00:(1, 7, 0, 0), 1.85:(1, 7, 7, 0), 2.38:(1, 7, 35, 35).

### 3.8 Model Prediction

In this work, we use a gradient boosted decision tree (GBDT) regressor to predict the target properties from our category-specific commutative algebra (CSCA) feature vectors. Gradient boosting transforms poor learners into strong predictors by creating an additive ensemble of shallow trees that are each fitted to the current model’s residuals under a squared error target.^25^ Although GBDT is a nonlinear model, the decision-making process remains interpretable because the input features are physically structured, category-resolved algebraic descriptors whose importance can be directly analyzed across scales and chemical groups.

We optimized the squared error loss function by implementing the gradient boosting regressor from Scikit-learn.^50^ The parameters of our model are displayed in Table 5. The targets were standardized, and all inputs were normalized. The data sets are divided as shown in Table 3; the validation split is not used for model selection because hyperparameters are fixed. We perform ten random splits and, within each split, train ten models with different seeds (100 training runs per data set) to reduce variance from random partitioning and initialization. We provide a solid, comparable evaluation to previous work by reporting RMSE, MAE, and the coefficient of determination, represented as *R* ^2^, as an average across runs on the 10% test set and, for completeness, on the pooled 20% (validation and test).

**Table 5:** Gradient Boosting Regressor Hyperparameters^a^

hyperparameter	value
max tree depth	7
max features per split	√*p*
min samples per leaf	1
min samples to split	2
learning rate	0.005
subsample	0.5
number of estimators	10,000

a Note: *p* denotes the number of input features.

## 4 Conclusion

Metal–organic frameworks (MOFs) are highly diverse and adaptable due to the mix of inorganic nodes and organic linkers, leading to a large surface area, tunable porosity, and a wide range of chemical compositions. However, identifying the structure–property relationships that govern adsorption and selectivity remains a significant challenge, especially when analyzing thousands of frameworks with varying compositions and architectures. Traditional experimental and computational techniques have limited capacity to generalize across the vast chemical and structural environment and grasp the intricate links between geometry and function.

This study introduced commutative algebra modeling and prediction, the first of its kind, to materials science. We developed category-specific commutative algebra (CSCA) machine learning to predict gas adsorption properties in MOFs. CSCA represents each framework using persistent facet ideals and f-vectors, calculated for chemically relevant atom groups within specific categories. This algebraic representation converts geometric connectivity into descriptors that are both rigorous and interpretable, thereby linking combinatorial algebra with material structure. The findings indicate that descriptors derived from low-dimensional facet ideals; dimensions 1 and 2 and f-vector components; dimensions 1, 2, and 3 consistently provide the most informative predictions for all four properties examined. In conclusion, CSCA machine learning furnishes a category-aware, algebraic representation of MOFs that yields accurate and interpretable predictors of gas adsorption. By computing facet-ideal statistics and *f*-vectors within and across chemically meaningful categories, the method links local composition with global connectivity in a way that is both rigorous and model-agnostic. The proposed commutative algebra approach has the potential to tackle a wide variety of other challenges in materials science.

## Supplementary Material



## Data Availability

All data sets originate from CoRE MOFs 2019 structures;^45^ Property calculations for each data set follow the approach outlined by Orhan et al.^44^ The code and data used in this study are provided at https://github.com/CSCA-MOFs/MOF-CSCA, with implementation details described in.^51^

## Notes

Additional details on model evaluation and data alignment; definitions of performance metrics, comparisons of benchmark data set sizes with prior studies, category resolved test performance statistics reported as mean ± standard deviation, and feature importance analyses across element-based categories for all four adsorption properties (PDF)
