# Supplementary material for: Commutative Algebra Modeling in Materials Science – A Case Study on Metal–Organic Frameworks (MOFs)
Source: J Chem Inf Model. 2026 Feb 17;66(5):2584–96. doi: 10.1021/acs.jcim.5c02817 (PMC12977046; doi:10.1021/acs.jcim.5c02817)
Supplement: Supplementary file 1 [file ci5c02817_si_001.pdf]

Supporting Information for Commutative Algebra Modeling in Materials Science – A Case Study on Metal-Organic Frameworks (MOFs)

Caleb Simiyu Khaemba [1], Hongsong Feng [2], Dong Chen [1],
Chun-Long Chen *[3] and Guo-Wei Wei†[1,4,5]
[1]Department of Mathematics,
Michigan State University, MI 48824, USA.
[2]Department of Mathematics and Statistics,
University of North Carolina at Charlotte, Charlotte, NC 28223, USA
[3]Physical Sciences Division, Pacific Northwest National Laboratory, Richland,
Washington 99354, USA
[4]Department of Electrical and Computer Engineering,
Michigan State University, MI 48824, USA.
[5]Department of Biochemistry and Molecular Biology,
Michigan State University, MI 48824, USA.

February 17, 2026

*Corresponding author: Chunlong Chen (chunlong.chen@pnnl.gov).
†Corresponding author: Guo-Wei Wei (weig@msu.edu).

S0.1 Performance Measures

We reported Root Mean Square Error (RMSE), Mean Absolute Error (MAE), and the coefficient of determination (R^2). Lower RMSE, lower MAE and higher R^2 indicated better performance. We used RMSE to capture overall prediction error with sensitivity to large deviations. We included MAE to provide a more interpretable measure of average error. We also reported R^2 to evaluate how much variance in the data was explained by our models.

$$\mathrm{RMSE} = \sqrt{\frac{1}{n}\sum_{i=1}^{n}(\hat{y}_i - y_i)^2}, \tag{1}$$

$$\mathrm{MAE} = \frac{1}{n}\sum_{i=1}^{n}|\hat{y}_i - y_i|, \tag{2}$$

$$R^2 = 1 - \frac{\sum_{i=1}^{n}(y_i - \hat{y}_i)^2}{\sum_{i=1}^{n}(y_i - \bar{y})^2}, \tag{3}$$

where n is the number of samples, y_i is the true value of sample i, $\hat{y}_i$ is the predicted value for sample i, and $\bar{y}$ is the mean of the true values.

S0.2 Benchmark Dataset Alignment

In this study we evaluated our model on four benchmark datasets describing the adsorption properties of MOFs: Henry's constants and uptake capacities for N_2 and O_2. The four datasets were adapted from the collection introduced by Orhan et al. [1], available through the public repository (`https://github.com/ibarishorhan/MOF-O2N2/tree/main/mofScripts`). Table S1 compares the size of our dataset with those of the three benchmark datasets. The Descriptor-based [1], MOF Transformer [2], and PM Transformer [3] models all rely on datasets from the same source, but the precise dataset details they used were not clearly described. We gathered and summarized the dataset sizes for each method in Table S1 for fairness and transparency.

Table S1: Comparison of dataset sizes used in this study and previous MOF models.

Property	CSCA	Descriptor-based[1]	MOF Transformer[2]	PM Transformer[3]
Henry's constant N_2	4744	4755	-	-
Henry's constant O_2	5036	5045	-	-
N_2 uptake	5132	5158	5286	5286
O_2 uptake	5241	5259	5286	5286

S0.3 Feature Importance

The distribution of feature importance across the eight element-based categories $C_a, \ldots, C_h$ shows how each descriptor family contributes to four adsorption characteristics, as illustrated in Figures

S1-S4. For a particular category, each tiny panel displays the aggregated random forest importance as a function of the filtering threshold α, with distinct curves for facet dimension 0, facet dimension 1, and f-vector dimensions 1, 2, and 3. Figures S1-S4 represent Henry's constants N_2, Henry's constant O_2, N_2 uptake, and O_2 uptake, respectively, while each subplot represents categories C_a through C_h and C_{all}. For each property, we trained a `RandomForestRegressor` with 100 trees, `max_features` set to `sqrt`, and a fixed seed for consistency. We utilized a 3-fold cross-validation repeated 5 times, with feature importances averaged over all folds and repeats.

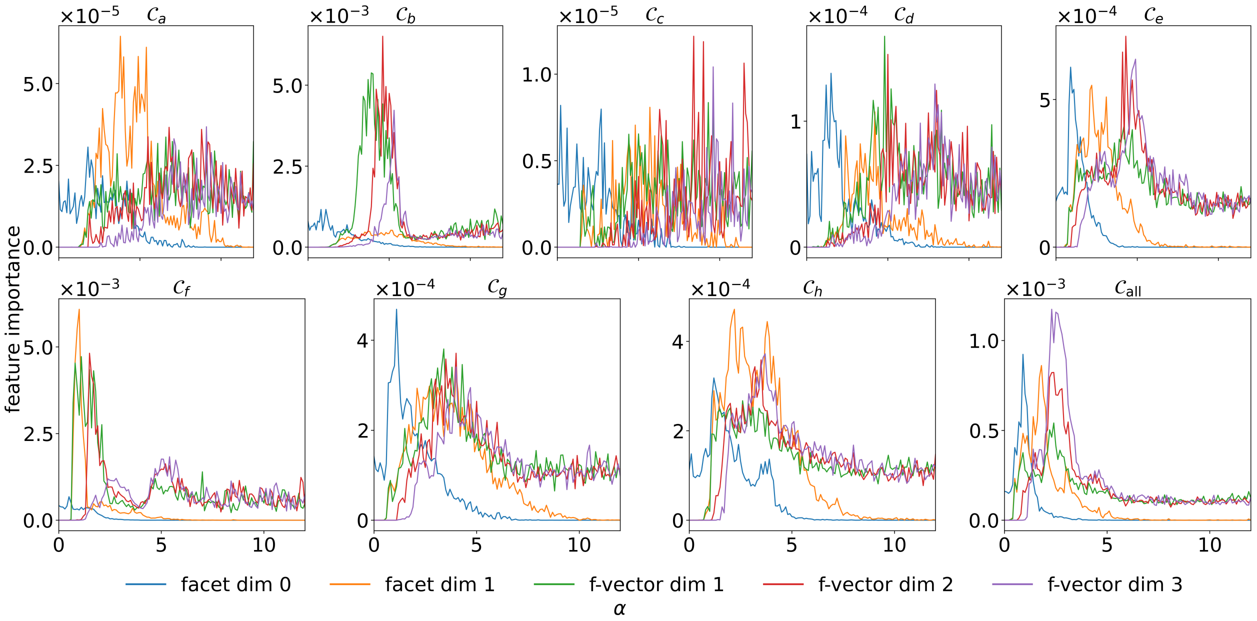

Figure S1: Distribution of feature importance across element-based categories $C_a, \ldots, C_h$ and C_{all} for Henry's constant of N_2. Each panel shows random forest importance as a function of the filtration threshold α, with curves corresponding to facet dimensions 0 and 1 and f-vector dimensions 1, 2, and 3.

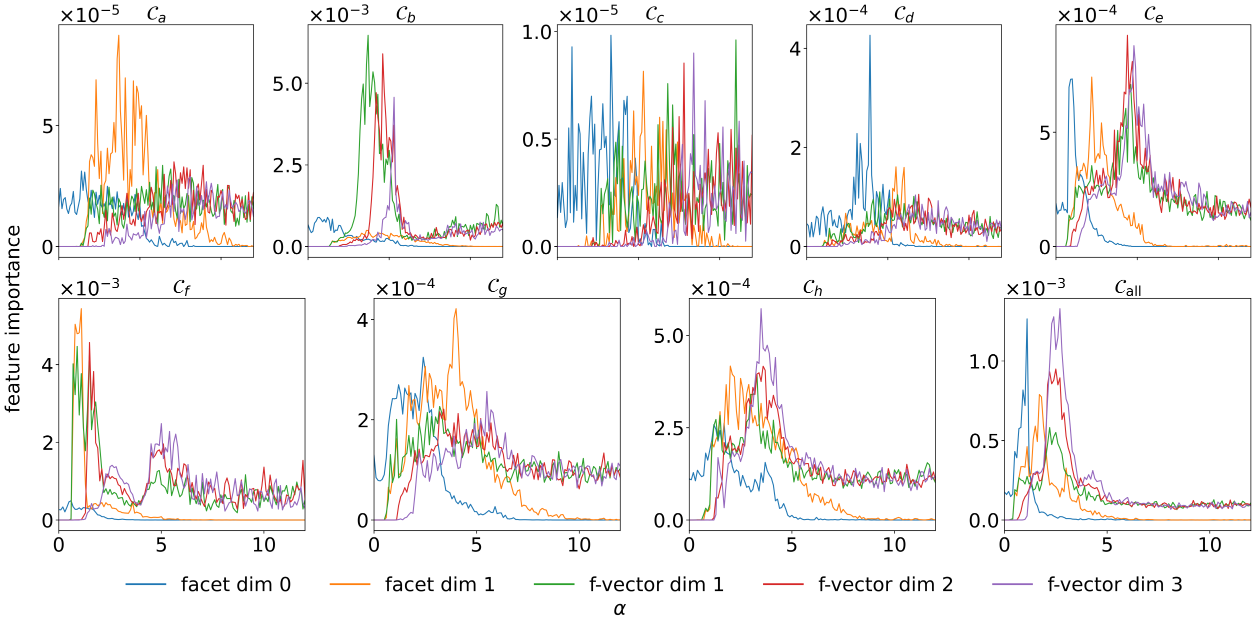

Figure S2: Distribution of feature importance across element-based categories $C_a, \ldots, C_h$ and C_{all} for Henry's constant of O_2. Each panel shows random forest importance as a function of the filtration threshold α, with curves corresponding to facet dimensions 0 and 1 and f-vector dimensions 1, 2, and 3

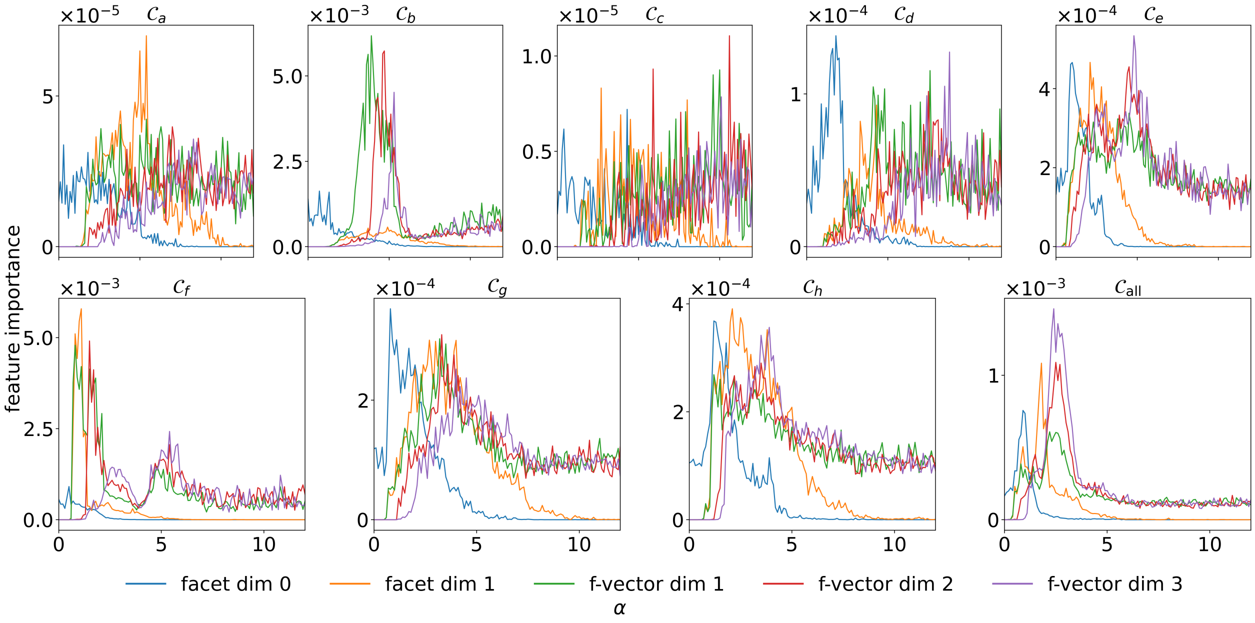

Figure S3: Distribution of feature importance across element-based categories $C_a, \ldots, C_h$ and C_{all} for N_2 uptake capacity. Each panel shows random forest importance as a function of the filtration threshold α, with curves corresponding to facet dimensions 0 and 1 and f-vector dimensions 1, 2, and 3

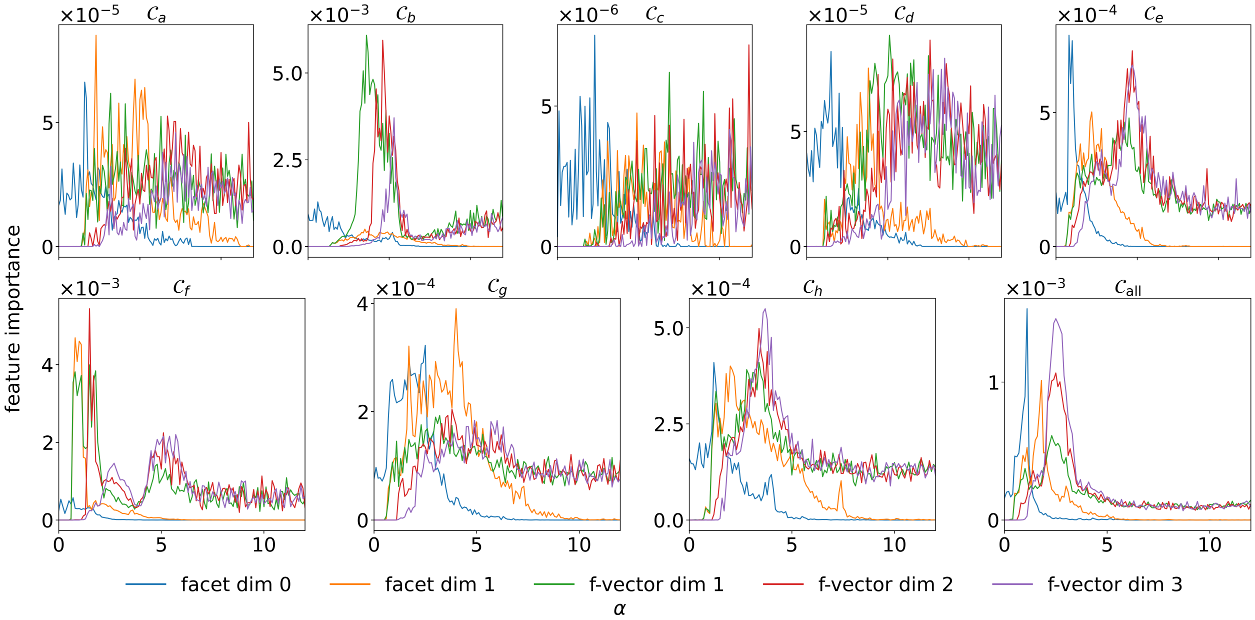

Figure S4: Distribution of feature importance across element-based categories $C_a, \ldots, C_h$ and C_{all} for O_2 uptake capacity. Each panel shows random forest importance as a function of the filtration threshold α, with curves corresponding to facet dimensions 0 and 1 and f-vector dimensions 1, 2, and 3.

Table S2 reports the mean and standard deviation of test performance over 10 independent random splits, each using an 80/10/10 train/validation/test partition. For each split, predictions are obtained by averaging over 10 Gradient Boosting models with different random seeds, resulting in 100 trained models per target property. Performance is evaluated using the coefficient of determination R^2, mean absolute error (MAE), and root mean squared error (RMSE), with higher R^2 and lower error indicating better predictive accuracy.

Table S2: CSCA test performance (mean ± std) over 10 random splits (80/10/10). For each split, predictions are averaged over 10 GBDT seeds (total 100 trained models per property).

**Dataset**	$\mathbf{R^2}$	**MAE**	**RMSE**
Henry's constant N_2	0.7725 ± 0.0282	$(5.2197 \pm 0.0234) \times 10^{-7}$	$(7.7054 \pm 0.0371) \times 10^{-7}$
Henry's constant O_2	0.8169 ± 0.0193	$(5.2970 \pm 0.0223) \times 10^{-7}$	$(8.0409 \pm 0.0393) \times 10^{-7}$
N_2 uptake	0.7780 ± 0.0156	0.05226 ± 0.00151	0.07692 ± 0.00260
O_2 uptake	0.8414 ± 0.0156	0.04723 ± 0.00279	0.07076 ± 0.00438

References

[1] Ibrahim B Orhan, Hilal Daglar, Seda Keskin, Tu C Le, and Ravichandar Babarao. Prediction of o2/n2 selectivity in metal–organic frameworks via high-throughput computational screening and machine learning. *ACS Applied Materials & Interfaces*, 14(1):736–749, 2021.

[2] Yeonghun Kang, Hyunsoo Park, Berend Smit, and Jihan Kim. A multi-modal pre-training transformer for universal transfer learning in metal–organic frameworks. *Nature Machine Intelligence*, 5(3):309–318, 2023.

[3] Hyunsoo Park, Yeonghun Kang, and Jihan Kim. Enhancing structure–property relationships in porous materials through transfer learning and cross-material few-shot learning. *ACS Applied Materials & Interfaces*, 15(48):56375–56385, 2023.
